# Immunoinformatics Design of a Broad-Spectrum Multi-Epitope Vaccine Targeting HA2 and M1 of H9N2 AIV

**DOI:** 10.3390/microorganisms14081617

**Published:** 2026-07-24

**Authors:** Jiashuang Ji, Yating Lin, Zijian Zhu, Kaixuan Yue, Yunhang Zhang, Wuchao Zhang, Baishi Lei, Wanzhe Yuan, Liwei Li, Kuan Zhao

**Affiliations:** 1 College of Veterinary Medicine, Hebei Agricultural University, Baoding 071001, China; jjs0820@126.com (J.J.); a664825313@163.com (Y.L.); z1795615095@163.com (Z.Z.); zhangyunhang@hebau.edu.cn (Y.Z.); zhangwuchao168@126.com (W.Z.); leibaishi2000@163.com (B.L.); yuanwanzhe@126.com (W.Y.); 2 Veterinary Biological Technology Innovation Centre of Hebei Province, Baoding 071000, China; 3 College of Life Science, Hebei Agricultural University, Baoding 071051, China; 2023044100132@stu.hebau.edu.cn; 4 Shanghai Veterinary Research Institute, Chinese Academy of Agricultural Sciences, Shanghai 200241, China

**Keywords:** H9N2, multi-epitope vaccine, immunoinformatics

## Abstract

H9N2 avian influenza virus (AIV) continues to mutate, leading to immunosuppression and secondary infections in poultry. Traditional inactivated vaccines mainly induce humoral immunity and have limited cross-protection efficacy against various subtypes of virus strains. In this study, we targeted the HA2 and M1 proteins of H9N2 as antigens and used immunoinformatics methods to design a broad-spectrum multi-epitope vaccine (MEV) that can simultaneously activate humoral and cellular immunity. Firstly, through systematic evolutionary analysis and sequence comparison, highly conserved amino acid sequence regions were selected from HA2 and M1 proteins. B-cell epitopes were predicted in the HA2 conserved sequence, and cytotoxic T lymphocyte (CTL) and helper T lymphocyte (HTL) epitopes were predicted in the M1 conserved sequence. Three candidate vaccines containing different epitope combinations were constructed. After secondary structure and physicochemical property comparisons, HM1 was determined as the optimal scheme. HM1 contains three B cell epitopes, two CTL epitopes, and three HTL epitopes, and was connected to chicken β-defensin at the N-terminus as a molecular adjuvant; a dendritic cell-targeting peptide was added at the C-terminus. The HM1 tertiary structure optimized by GalaxyRefine met the standards of a reliable model. The molecular docking results indicated that HM1 can form stable binding with chicken TLR2, TLR4, MHC I, and MHC II molecules, with binding free energies of −7.1 kcal/mol and −6.1 kcal/mol, respectively, and can form multiple hydrogen bonds and salt bridges. Normal mode analyses revealed that the HM1–TLR complex exhibits favorable dynamic properties at the computational level. The immune simulation prediction results showed that after vaccination with HM1, specific antibodies can be induced, B cells, helper T cells, and cytotoxic T cells can be activated, and IFN-γ and IL-2 can be secreted. In summary, the HM1 designed based on the conserved regions of HA2 and M1 proteins has good physicochemical stability and immunogenicity, providing a theoretical basis for the development of broad-spectrum and highly effective H9N2 vaccines.

## 1. Introduction

Avian influenza (AI), caused by the avian influenza virus (AIV), is a highly contagious viral disease that mainly infects all types of domestic poultry and wild birds, and even breaks through the interspecies barrier to directly infect mammals and humans [1]. AIV is typically classified into low-pathogenic AIV (LPAIV), which has a relatively low incidence rate, and highly pathogenic AIV (HPAIV), which can lead to severe clinical symptoms and high mortality. In recent years, multiple subtypes of AIV have become prevalent worldwide [2]. Among them, H9N2, as an LPAIV, has been widespread in various countries since its first isolation in Wisconsin, USA, in 1966 [3]. It often occurs in mixed or secondary infections caused by other pathogens, causing slow growth, decreased egg production, and immunosuppression in poultry, resulting in severe economic losses to the global poultry industry.

H9N2 belongs to the genus *Influenzavirus A* within the family *Orthomyxoviridae*, and is a negative-sense single-stranded RNA virus with a segmented genome [4,5,6]. It has eight independent genomic segments that encode multiple structural proteins, including hemagglutinin (HA), neuraminidase (NA), matrix protein 1 (M1), matrix protein 2 (M2), and nucleoprotein (NP) [7,8]. Due to the lack of proofreading activity of the viral RNA-dependent RNA polymerase, AIV tends to accumulate mutations during replication. In recent years, H9N2 has undergone rapid evolution, and the antigenic drift among different strains has become increasingly significant, leading to a gradual decline in the protective efficacy of existing vaccines against emerging strains [9]. At present, the research focus on H9N2 vaccines is on enhancing their immune efficacy and broad-spectrum protective capacity [10]. Using naringenin as an adjuvant has significantly boosted the humoral and cellular immunity of inactivated H9N2 vaccines and reduced the tissue pathological damage caused by the virus [11]. The construction of a universal influenza vaccine for H9N2/H7N9 using ferritin self-assembled nanoparticles and a recombinant *Salmonella* delivery system has successfully induced potent cellular and humoral immunity and demonstrated a certain degree of cross-protection [12]. Additionally, research on the immune mechanism of inactivated H9N2 vaccines has been continuously advanced. Comparisons among five commercial inactivated H9N2 vaccines have revealed that they trigger immune responses by activating the TLR-5/TLR-7 pathways, with significant differences in protective efficacy among vaccines formulated with different adjuvants [9].

Meanwhile, the application of novel vaccine technologies has provided new strategies for H9N2 vaccine research [13]. Multi-epitope vaccines (MEVs) offer a key advantage in that they utilize the minimal immunogenic fragments to induce effective immune responses against specific pathogens [14]. By linking multiple antigenic epitopes, MEVs can be used to prevent various viruses or different serotypes of the same virus [15]. Compared with traditional vaccines, MEVs do not require microbial cultivation and have the advantages of low cost, high specificity, high safety, ease of preservation and modification [16,17,18]. MEVs contain B-cell epitopes, cytotoxic T-lymphocyte (CTL) epitopes and helper T-lymphocyte (HTL) epitopes, which can simultaneously induce humoral and cellular immune responses, with stronger immunogenicity and broader coverage [19]. In addition, MEVs can focus the immune response on conserved epitopes, improving the cross-protection efficacy against multiple pathogens [20]. With the inclusion of adjuvant components, MEVs can further potentiate immunogenicity and induce long-lasting immunity.

HA is a key multifunctional protein of AIV that mediates viral binding to the host receptors and is cleaved into HA1 and HA2 subunits [21]. HA1 forms a highly variable spherical head, containing the major receptor binding sites and subtype-specific neutralizing epitopes, making it a critical antigen for inducing protective antibody responses [22]. HA2 forms the majority of the stem domain and is highly conserved among subtypes. It plays an essential role in mediating the fusion of the virus with the endosomal membrane and contains broad-spectrum neutralizing epitopes, which can induce cross-protective immunity against different subtype AIV and may have significant potential for developing universal vaccines. M1 is a highly conserved structural protein of AIV, accounting for 30–40% of the total viral protein. It participates in multiple life cycle processes such as virus assembly, budding, and nuclear export of the ribonucleoprotein complex. It is one of the main target proteins recognized by host CTLs. Vaccines based on M1 can induce strong CD8^+^ T cell responses and provide cross-protection against homologous and heterologous influenza viruses. The high sequence conservation of M1 across different AIV subtypes can stimulate a wide range of T cell immune responses, positioning it as an important candidate antigen for developing broad-spectrum influenza vaccines. Although HA1 plays an important role in inducing subtype-specific immune responses, its high variability limits the development of broad-spectrum vaccines. In contrast, HA2 and M1 are highly conserved among the same subtypes of AIV and can induce broad cross-protective responses at both the humoral and cellular immune levels. Therefore, HA2 and M1 are ideal molecular targets for designing broad-spectrum MEVs against H9N2.

**Figure 1:**
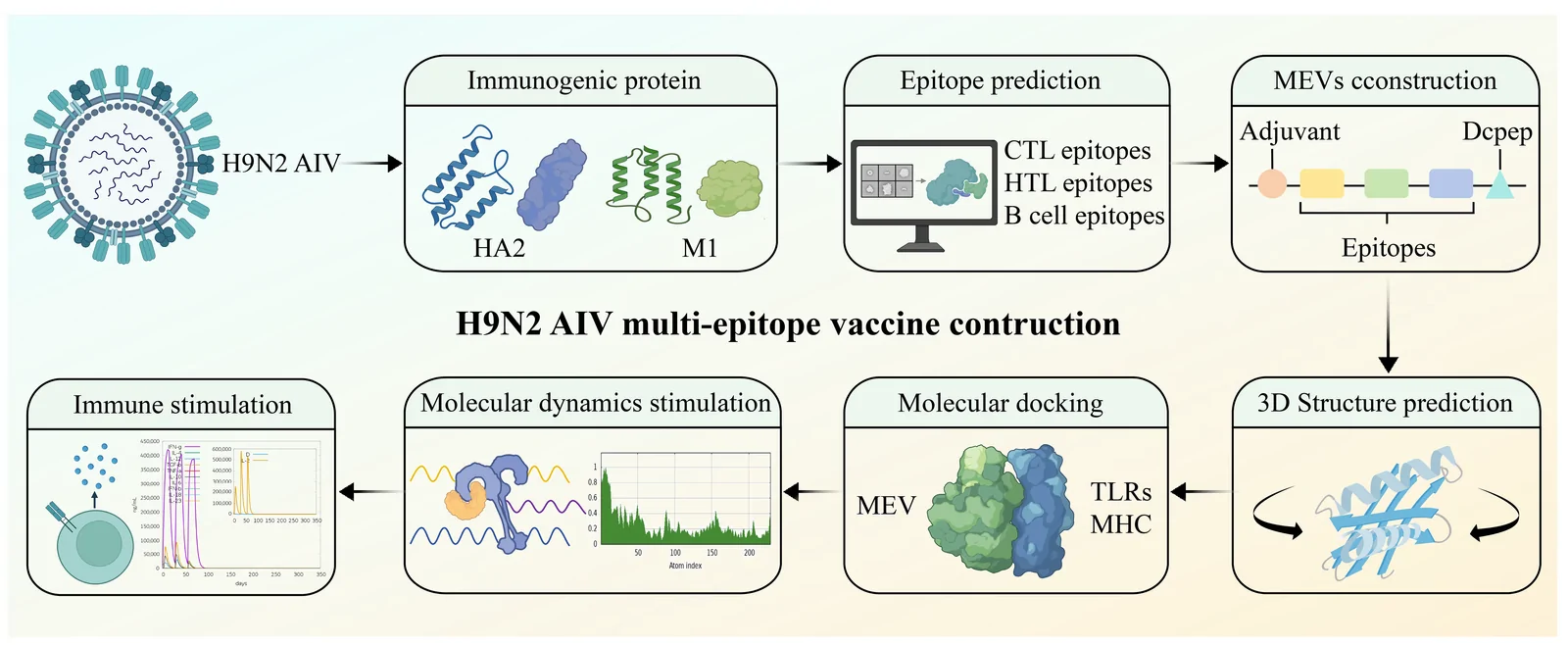
Flowchart for constructing a multi-epitope vaccine against H9N2.

Based on the highly conserved characteristics of HA2 and M1, this study employed immunoinformatics methods to predict B-cell epitopes of HA2, CTL epitopes and HTL epitopes of M1, and designed a broad-spectrum MEV for H9N2. The binding ability of the MEV to TLRs and MHC molecules, as well as is potential to induce immune responses, was evaluated through molecular docking, normal-mode analyses and immune response simulation. This study can provide a certain theoretical basis for the development of safe, broad-spectrum and highly effective novel H9N2 vaccines. The overall workflow used in this study is shown in Figure 1.

## 2. Materials and Methods

### 2.1. Genetic Evolution Analysis and Conserved Sequence Screening

Fifty H9N2 strains were downloaded from the NCBI GenBank database (https://www.ncbi.Nlm.nih.gov/, accessed on 5 March 2026). These strains belong to three main genetic lineages: G1-like, Y280-like and F98-like. The geographical sources cover China (more than ten provinces) and the United States. The collection years range from 1966 to 2023. Each contains the amino acid sequences of the HA and M proteins. Detailed information on all strains and their corresponding relationships was listed in Table S1. Phylogenetic analysis and sequence similarity comparisons for HA and M sequences were performed separately using MEGA 7.0.

### 2.2. Prediction of Sequence Antigenicity and T and B Cell Epitopes

The antigenicity of all conserved sequences was predicted using ANTIGENpro (https://scratch.proteomics.ics.uci.edu/, accessed on 6 March 2026), with the threshold parameter set to 0.6. For HA2 conserved sequences exhibiting antigenicity, linear B-cell epitopes were predicted using ABCpred (https://webs.iiitd.edu.in/raghava/abcpred/, accessed on 6 March 2026), with the threshold and window length set to 0.60 and 16 mer, respectively. Previous studies have shown that the B-F allele of chickens has similar immune response functions in antigen presentation as the human class I alleles [23]. Based on this, we followed the methods reported in the previous literature and selected the optimal human allele combination suitable for chickens. For M1 conserved sequences exhibiting antigenicity, CTL epitopes were predicted using NetMHCcons 1.1 (https://services.healthtech.dtu.dk/services/NetMHCcons-1.1/, accessed on 6 March 2026), with the amino acid length set at 9, and the HLA-B*40:06, HLA-B*41:04 and HLA-B*41:03 alleles were selected. HTL epitopes were predicted by NetMHCIIpan 4.3 (https://services.healthtech.dtu.dk/services/NetMHCIIpan-4.3/, accessed on 6 March 2026) server, with the epitope peptide length set at 15 mer. The selected alleles included DRB1*1482, DRB1*1366, DRB1*1310, and DRB1*1445 [23]. The screening threshold for strong binding epitopes was set at %Rank < 0.5, and the threshold for weak binding epitopes was set at %Rank < 2.0. All the predicted B-cell epitopes, CTL epitopes and HTL epitopes were, respectively, predicted for their sensitization and toxicity using AllerTOP v2.0 (https://ddg-pharmfac.net/allertop_test/, accessed on 10 March 2026) and ToxinPred 2 (https://webs.iiitd.edu.in/raghava/toxinpred2/, accessed on 10 March 2026). Furthermore, the amino acid sequences of all epitopes were compared with the corresponding protein sequences of 50 strains. The number of strains in which each epitope sequence was completely identical among the 50 strains was counted, and the conservation percentage was calculated.

### 2.3. Multiple Epitope Antigens Design

Based on the prediction results of MHC binding affinity, allergenicity, toxicity and MHC allele coverage, the most optimal epitopes were selected for vaccine construction. According to the different types and quantities of antigenic epitopes, three MEV constructs were designed. Since individual epitopes do not have sufficient immunogenicity, adjuvants are required to enhance the immune response [24]. Chicken β-defensin is an important innate immune effector molecule in chickens. Studies have shown that it can enhance the immune response induced by DNA vaccines as a molecular adjuvant [25], suggesting its potential application in protein vaccines as well. Therefore, the mature peptide segment of chicken β-defensin was introduced at the N-terminus as a molecular adjuvant. CTL, HTL, and B-cell epitopes were linked using AAY, GPGPG, and KK linkers, respectively. Different epitopes were connected using a linker (GPGPGLRMKLPKS). Furthermore, studies have shown that integrating dendritic cell-targeting peptides into vaccine antigens can significantly enhance the efficiency of antigen uptake by dendritic cells and induce stronger antibody and cellular immune responses [26]. Therefore, dendritic cell-targeting peptides (FYPSYHSTPQRP) were added to the C-terminal of MEV.

The secondary structures of MEVs were predicted using PSIPRED (http://bioinf.cs.ucl.ac.uk/psipred/, accessed on 15 March 2026). The physicochemical properties of MEVs, HA2, M1, and HA2-M1 fusion protein (HMf) were analyzed using Expasy ProtParam (https://web.expasy.org/protparam/, accessed on 15 March 2026). Allergenicity was assessed using AllerTOP v2.0 (https://ddg-pharmfac.net/allertop_test/, accessed on 15 March 2026), and toxicity was evaluated using ToxinPred 2 (https://webs.iiitd.edu.in/raghava/toxinpred2/, accessed on 15 March 2026). Antigenicity was predicted using ANTIGENpro (https://scratch.proteomics.ics.uci.edu/, accessed on 15 March 2026), while solubility in the *E. coli* expression system was predicted using SOLpro (https://scratch.proteomics.ics.uci.edu/explanation.html, accessed on 15 March 2026).

### 2.4. Tertiary Structure Modeling and Validation

The tertiary structure of MEV was predicted by I-TASSER (https://zhanggroup.org/I-TASSER/, accessed on 18 March 2026). The structure was optimized using GalaxyRefine (https://galaxy.seoklab.org/, accessed on 19 March 2026), and the best model was selected as the optimized tertiary structure based on a comprehensive evaluation of refinement-specific quality metrics, including GDT-HA, RMSD, MolProbity score, and Clash score. The Ramachandran plot was generated by PROCHECK (https://saves.mbi.ucla.edu/, accessed on 19 March 2026) within the SAVERv6.0 to analyze the amino acids and overall structure of the optimized model, and to determine its stereochemical quality. The Z-score of the model was calculated using ProSA-Web (https://prosa.services.came.sbg.ac.at/prosa.php, accessed on 19 March 2026) to assess its overall folding quality.

### 2.5. Molecular Docking Analysis

To determine whether MEV can effectively bind to immune receptors, molecular docking between the tertiary structure of MEV and chicken TLR2 (UniProt ID: Q9DD78), TLR4 (UniProt ID: C4PCF3), MHC I (UniProt ID: P15979), and MHC II (PDB ID: 6KVM) was performed using the GRAMM (https://gramm.compbio.ku.edu/, accessed on 26 March 2026). The interface analysis of the optimal docking complex was conducted using PDBePISA (https://www.ebi.ac.uk/pdbe/pisa/, accessed on 27 March 2026) to calculate the buried surface area, binding free energy, hydrogen bonds and salt bridges. COCOMAPS 2.0 (https://aocdweb.com/BioTools/cocomaps2/, accessed on 27 June 2026) was used to identify and quantify various types of atomic interactions at the complex interface, quantitatively analyze the buried surface area, and provide comprehensive characterization and visualization of the molecular interactions at the interface of the biomolecular complexes [27]. The docking results were visualized with PyMOL 3.0.3. The two-dimensional images were presented using the visualization tool Ligplot+ 2.3.1.

### 2.6. iMODS-Based Normal Mode Analysis

Normal mode analysis based on an elastic network model was performed on the HM1–TLR2 and HM1–TLR4 complexes using the iMODS web server (https://imods.iqf.csic.es/, accessed on 11 April 2026) [28,29] to evaluate their intrinsic flexibility and collective motions under the optimal docking models. To further characterize the dynamic properties of the docking complexes, additional parameters, including B-factors, deformability, covariance, variance, elastic network models, and eigenvalues were assessed [30].

### 2.7. Immune Simulation

To evaluate the immunogenicity and immune response characteristics of MEV, computerized immune simulations were performed using the C-ImmSim (https://kraken.iac.rm.cnr.it/C-IMMSIM, accessed on 15 April 2026) [31]. The simulation was carried out in three injections, with an injection interval of 4 weeks each. The total simulation steps were set at 1050, with a standard time step of 8 h for each injection, and the individual injection time steps were 1, 84, and 168, respectively. All other parameters were set to their default values. The analyzed immune parameters include the titers of immunoglobulins (IgM, IgG1, IgG2, IgG), the number of antibody-secreting cells, B cell subsets (total B cells, memory B cells, activated B cells), helper T cell subsets (total TH cells, activated TH cells), cytotoxic T cell subsets (activated TC cells, resting TC cells), and cytokine concentrations (IFN-γ, IL-2).

## 3. Results

### 3.1. Phylogenetic and Conserved Sequence Analysis of HA and M

**Figure 2:**
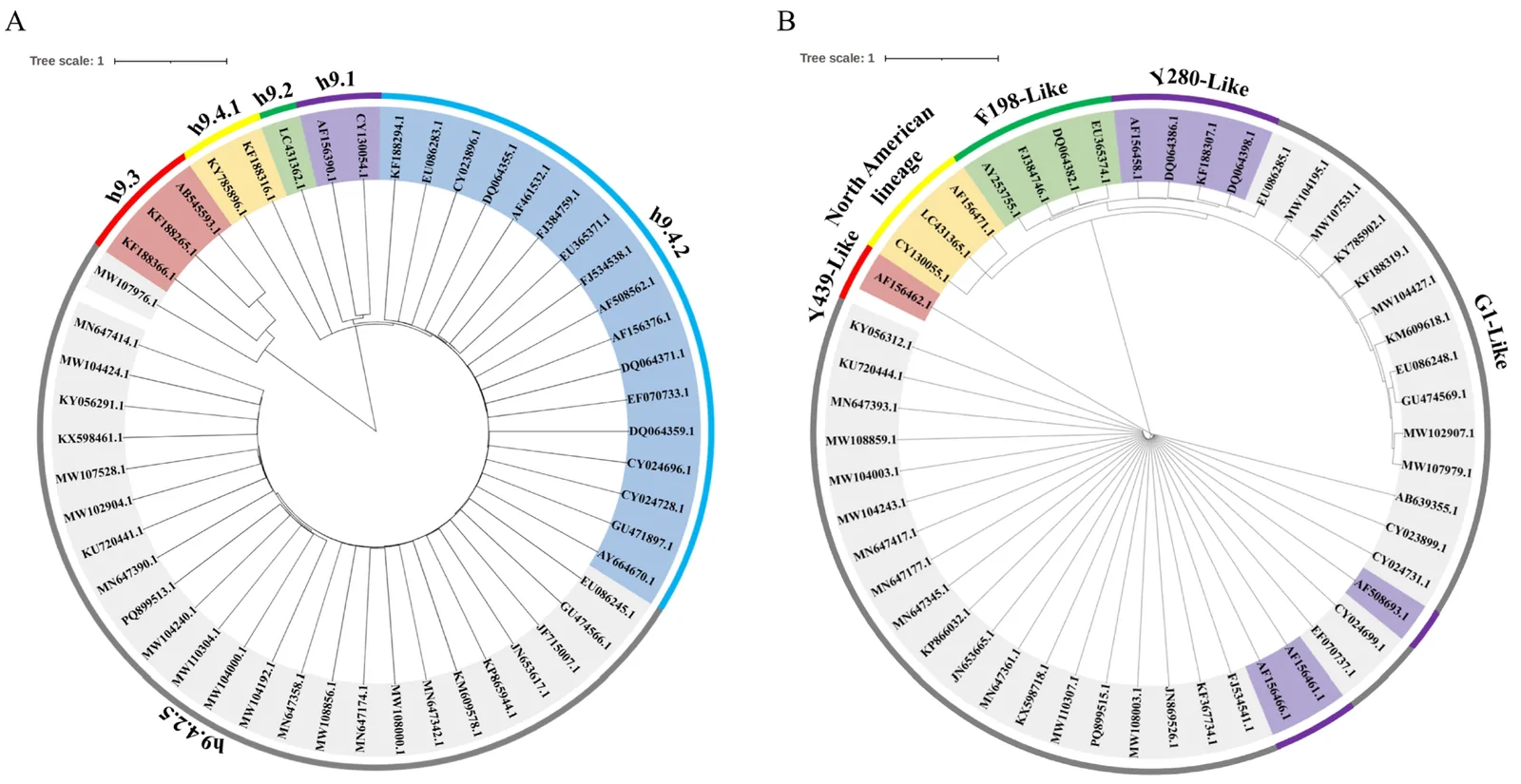
Phylogenetic analysis of H9N2 epidemic strain. (**A**) Phylogenetic tree of H9N2 HA. (**B**) Phylogenetic tree of H9N2 M. The trees were generated using MEGA 7.0 software and the Neighbor-joining method with 1000 bootstrap replicates.

To analyze the genetic diversity of the selected AIV strains, phylogenetic trees were constructed based on the amino acid sequences of the HA and M genes, respectively. The evolutionary analysis of the HA gene divided the 50 H9N2 strains into 6 main branches, among which branch h9.4.2.5 contained most of the domestic epidemic strains isolated in recent years (Figure 2A). The phylogenetic analysis of the M gene revealed that the selected strains were mainly divided into three branches: G1-like, Y280-like, and F98-like (Figure 2B). The above results indicated that the 50 selected strains cover the main genetic branches of H9N2, providing a reliable sequence basis for the subsequent design of MEVs. Based on amino acid sequence similarity comparison results, a total of 6 conserved sequences in the HA2 and M1 proteins were selected. These sequences were submitted to the ANTIGENpro for antigenicity prediction. Based on the antigenicity scores, the amino acid intervals of 10–68, 70–138 in the HA2 protein, and the amino acid intervals of 38–139 and 159–221 in the M1 protein were selected for subsequent epitope prediction. Detailed information is provided in Table 1.

### 3.2. Selection of Dominant Antigen Epitopes

Since HA2 contains broad-spectrum neutralizing epitopes, it can provide cross-protection against different subtypes of AIV. Linear B-cell epitopes in its conserved sequences were predicted using ABCpred. A total of 10 epitopes with strong binding affinity were identified, with scores ranging from 0.85 to 0.90. Detailed information was shown in Table 2.

The M1 protein is one of the major target proteins recognized by host T cells during AIV infection and primarily induces cellular immune responses. Using NetMHCcons 1.1, 4 CTL epitopes with strong binding affinity (IC50 < 500 nM) were identified from the conserved sequences of M1. The results were summarized in Table 3. Using the NetMHCIIpan 4.3 for HTL epitope prediction, 13 epitopes with immunogenic potential were predicted based on the Rank (%) value (Table 4). All epitopes were predicted to be non-allergenic and non-toxic by AllerTOP v2.0 and ToxinPred 2, respectively.

### 3.3. Design of Multi-Epitope Antigen Schemes

**Figure 3:**
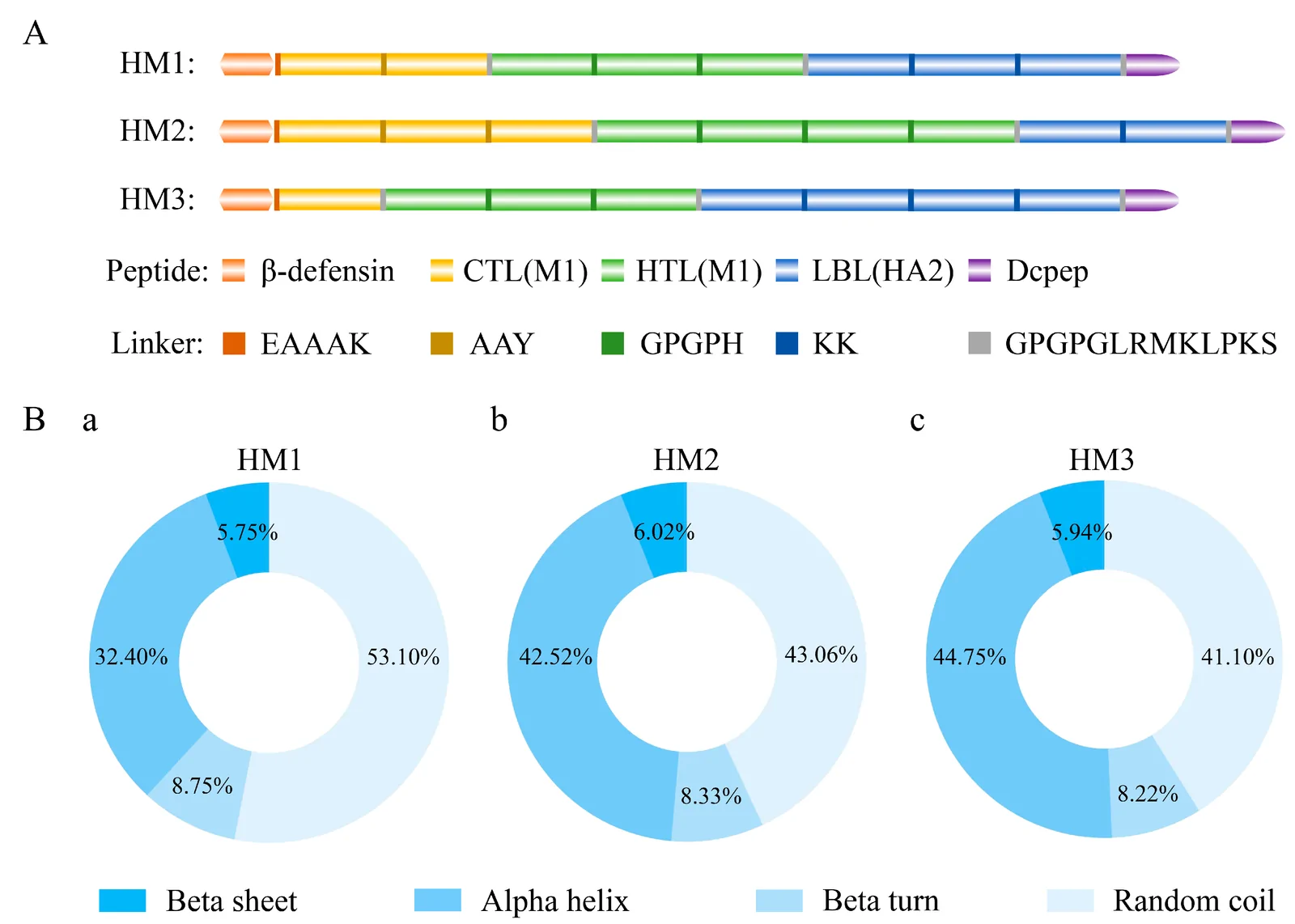
(**A**) Schematic diagram of vaccine constructs and secondary structure prediction of (**B**, a) HM1, (**B**, b) HM2, and (**B**, c) HM3.

The epitopes with the best prediction results and broad allele coverage were selected. Three MEV constructs were designed. HM1 contains 3 B-cell epitopes (2-1, 2-2, and 2-3), 2 CTL epitopes (2-1 and 2-3), and 3 HTL epitopes (2-2, 2-5, and 3-3), with a preference for B/T cell co-recognized epitopes. HM2 contains 2 B-cell epitopes (2-1 and 2-2), 3 CTL epitopes (2-1, 2-3, and 3-1), and 4 HTL epitopes (2-2, 2-5, 3-3, and 3-6), covering more T-cell epitopes with a relatively longer sequence. HM3 contains 4 B-cell epitopes (1-1, 2-1, 2-2, and 2-3), 1 CTL epitope (2-1), and 3 HTL epitopes (2-2, 2-5, and 3-3), favoring epitopes with strong immunogenicity. In each construct, CTL, HTL, and B-cell epitopes were fused using AAY, GPGPG, and KK linkers, respectively, while different epitopes were connected by a universal linker (GPGPGLRMKLPKS). To enhance the immunogenicity of MEVs, chicken beta-defensin was fused at the N-terminus as a molecular adjuvant, and a dendritic cell-targeting peptide (FYPSYHSTPQRP) was added at the C-terminus to improve antigen presentation efficiency. The structures of the three MEVs are shown in Figure 3A, with lengths of 287 aa, 352 aa, and 363 aa, respectively. AllerTOP v2.0 and ToxinPred 2 were used to predict the three schemes, and the results indicated that none of the three schemes had allergenicity or toxicity.

### 3.4. Prediction of Secondary Structure and Physicochemical Properties

To further select the optimal vaccine candidate, the secondary structure characteristics and physicochemical properties of the three MEVs were comprehensively compared. Secondary structure prediction showed that the proportion of random coils and β-turns in HM1 was 61.85%, significantly higher than that of HM2 (51.39%) and HM3 (49.32%), indicating that HM1 had the optimal potential for B-cell epitope exposure (Figure 3B).

**Figure 4:**
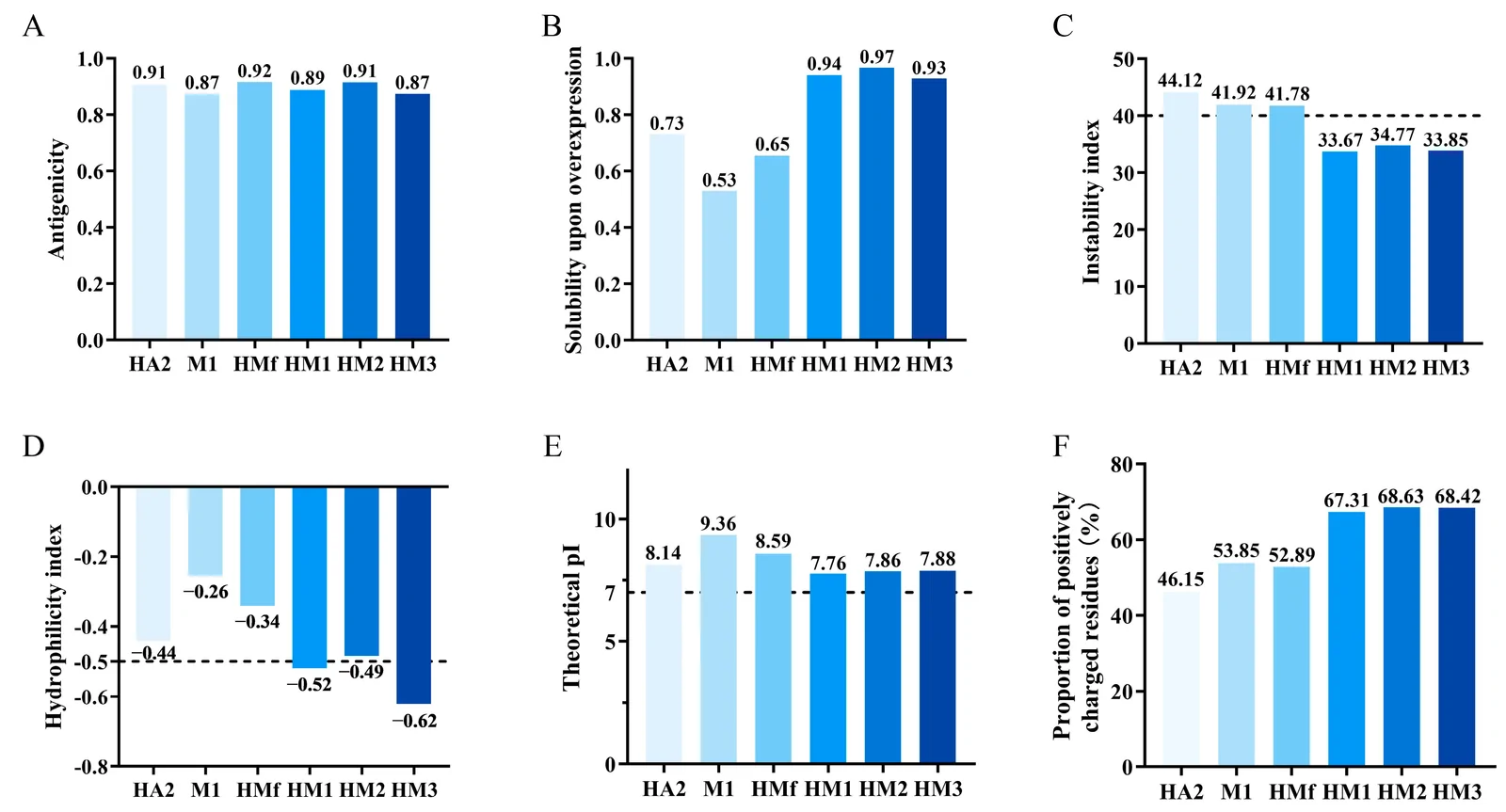
Prediction and analysis of the physicochemical properties of MEVs and natural proteins, including (**A**) antigenicity, (**B**) solubility upon overexpression, (**C**) instability index, (**D**) hydrophilicity index, (**E**) theoretical pI, and (**F**) proportion of positively charged residues.

The physicochemical property prediction results are shown in Figure 4. The antigenicity scores of all three constructs were comparable to those of HA2, M1, and HMf, suggesting that all three possessed strong immunogenic potential (Figure 4A). Solubility prediction results showed that the solubility scores of the three constructs in *E. coli* were all above 0.90, considerably higher than HA2, M1, and HMf, indicating that all three constructs had better solubility in the Escherichia coli system and had better solubility than the natural protein (Figure 4B). Instability index prediction showed that the instability coefficients of HA2, M1, and HMf were all greater than 40, indicating unstable proteins. In contrast, all three constructs had instability indices lower than 40, and HM1 had the lowest value (33.67), indicating the best in vitro stability potential (Figure 4C). Hydropathy index prediction showed that the predicted hydropathy indices of the three constructs were all negative, below the hydrophilic threshold of 0, indicating good hydrophilicity, with HM1 being −0.52, the most hydrophilic among all proteins (Figure 4D). Isoelectric point prediction showed that the pI values of the three constructs ranged from 9.76 to 9.88, within the ideal range (Figure 4E). The positive charge residue proportion results showed that the proportion of positive charge residues in three constructs was all greater than 65%, higher than HA2, M1, and HMf, indicating that the three constructs all had a high positive charge density, which is conducive to electrostatic interaction with negatively charged cell membranes (Figure 4F).

By comprehensively comparing the secondary structure and physicochemical properties, HM1, which exhibited the optimal secondary structure exposure characteristics and balanced physicochemical properties, was selected as the preferred vaccine candidate for subsequent immunoinformatics analysis.

### 3.5. Prediction and Optimization of HM1 Tertiary Structure

**Figure 5:**
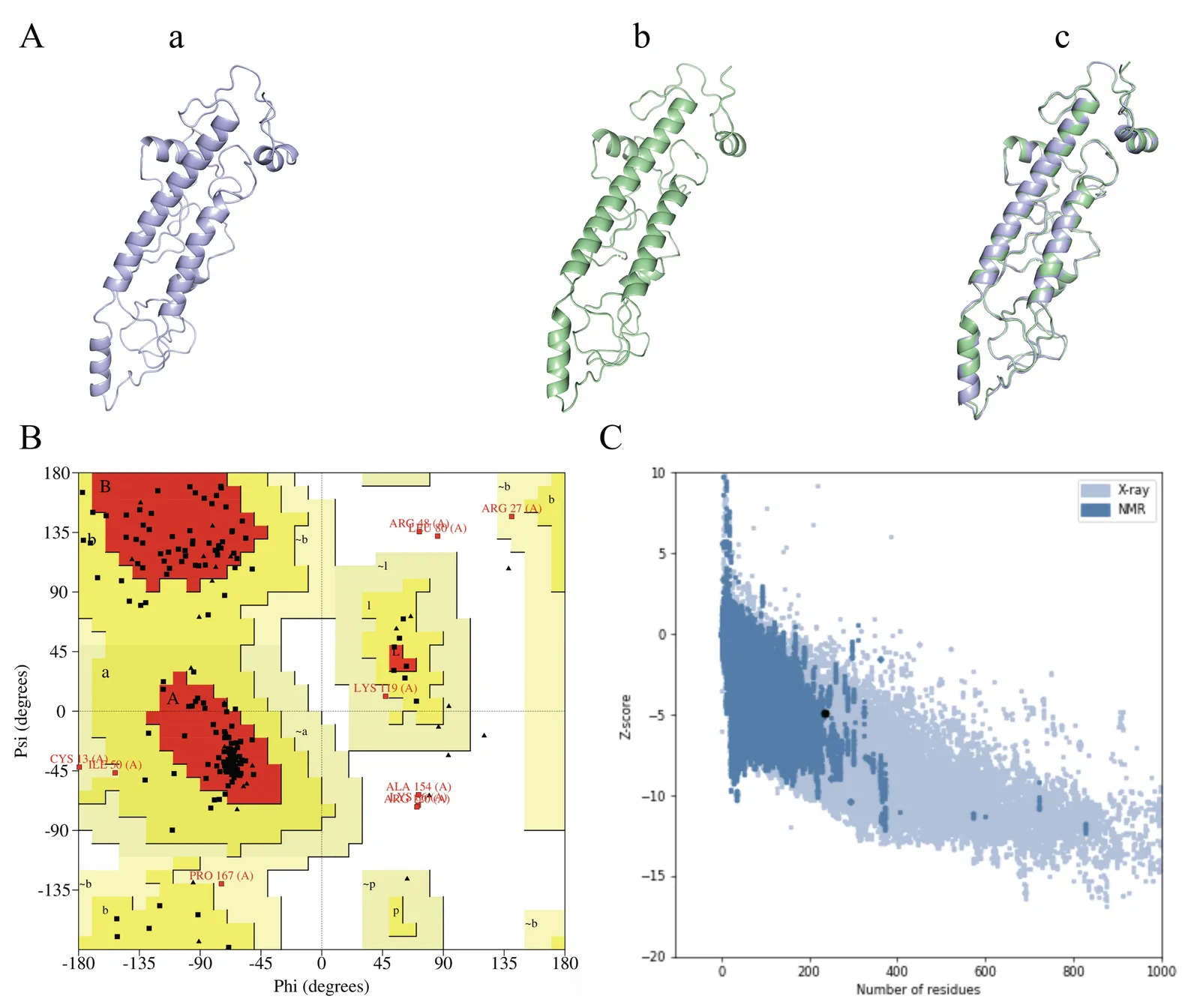
Structure prediction and validation of vaccine construct HM1. (**A**, a) The tertiary structure constructed by I-TASSER. (**A**, b) The tertiary structure optimization by Galaxy Refine server. (**A**, c) The overlap situation of the models before and after optimization. (**B**) Ramachandran plot. The triangles denote glycine residues, and the squares represent other standard amino acid residues. The regions are categorized as most favoured (A, B, L), additional allowed (a, b, l, p), and generously allowed (~a, ~b, ~l, ~p), as shown in the figure. (**C**) Z-score graph (overall quality).

The tertiary structure of HM1 was modeled using I-TASSER and visualized with PyMOL (Figure 5(Aa)). The initial model was optimized using GalaxyRefine to improve its quality, resulting in five refined 3D models. The selected best-optimized model exhibited a GDT-HA value of 0.8957, an RMSD value of 0.591, a MolProbity score of 2.455, and a clash score of 18.9 (Figure 5(Ab)). Compared with the initial model, the structural rationality and stereochemical quality of the optimized model were significantly improved. The overlap of the models before and after optimization is shown in Figure 5(Ac). Ramachandran plot analysis indicated that 79.0% of the residues in the optimized model were in the most favorable regions, 16.0% in the additional allowed regions, 2.2% in the generously allowed regions, and 2.8% in the disallowed regions. The residues located in the forbidden region are specifically ARG48, LEU80, LYS86, ARG120, and ALA154 (Figure 5B). Compared with the pre-optimized model, the proportion of residues in the most favorable regions increased significantly, indicating that the stereochemical quality of the optimized model was effectively improved. The quality of the optimized model was evaluated by ProSA-Web, and its Z-score was −4.98 (Figure 5C), which fell within the Z-score range of naturally determined protein structures, suggesting that the model has overall stereochemical and energy characteristics similar to those of natural proteins. The above data collectively indicate that the tertiary structure of HM1 optimized by GalaxyRefine is a reliable model, which can be used for subsequent molecular docking and functional mechanism studies.

### 3.6. Molecular Docking of HM1 with TLRs and MHC Molecules

**Figure 6:**
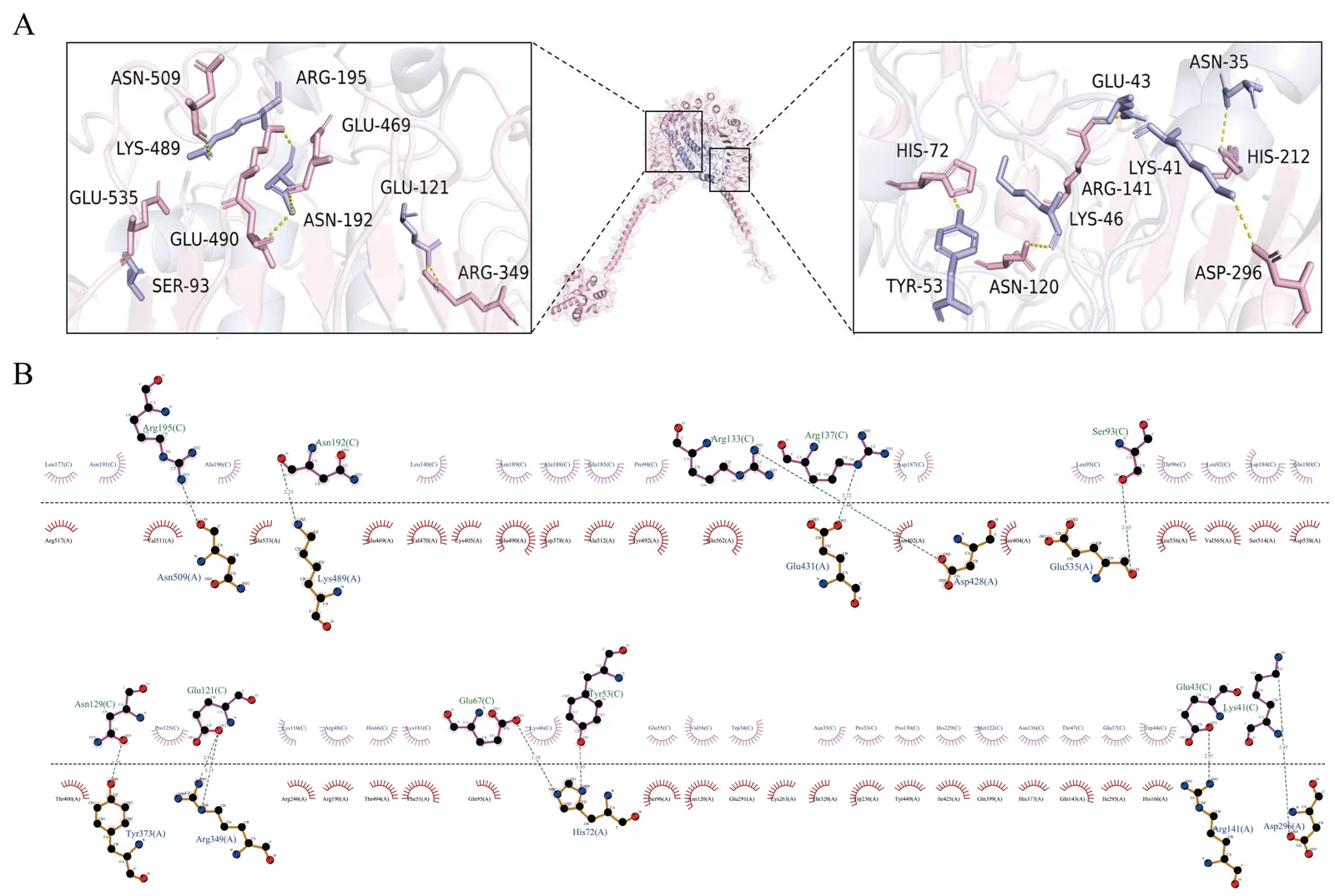
Molecular docking between HM1 and TLR2. (**A**) Diagram of the docking model of HM1–TLR2 complex. (**B**) Used the visualization tool Ligplot+ to display two-dimensional images.

**Figure 7:**
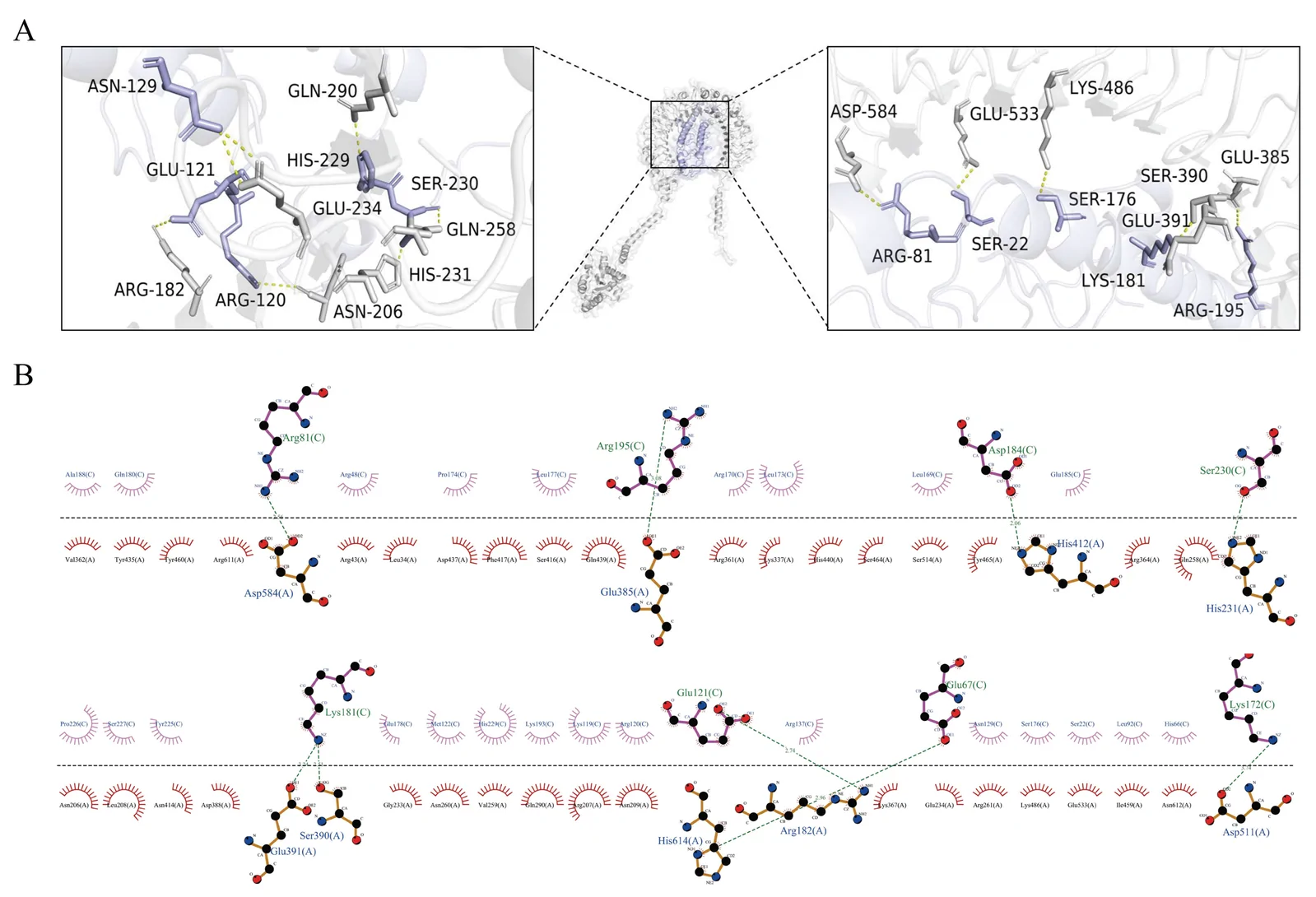
Molecular docking between HM1 and TLR4. (**A**) Diagram of the docking model of HM1–TLR4 complex. (**B**) Used the visualization tool Ligplot+ to display two-dimensional images.

**Figure 8:**
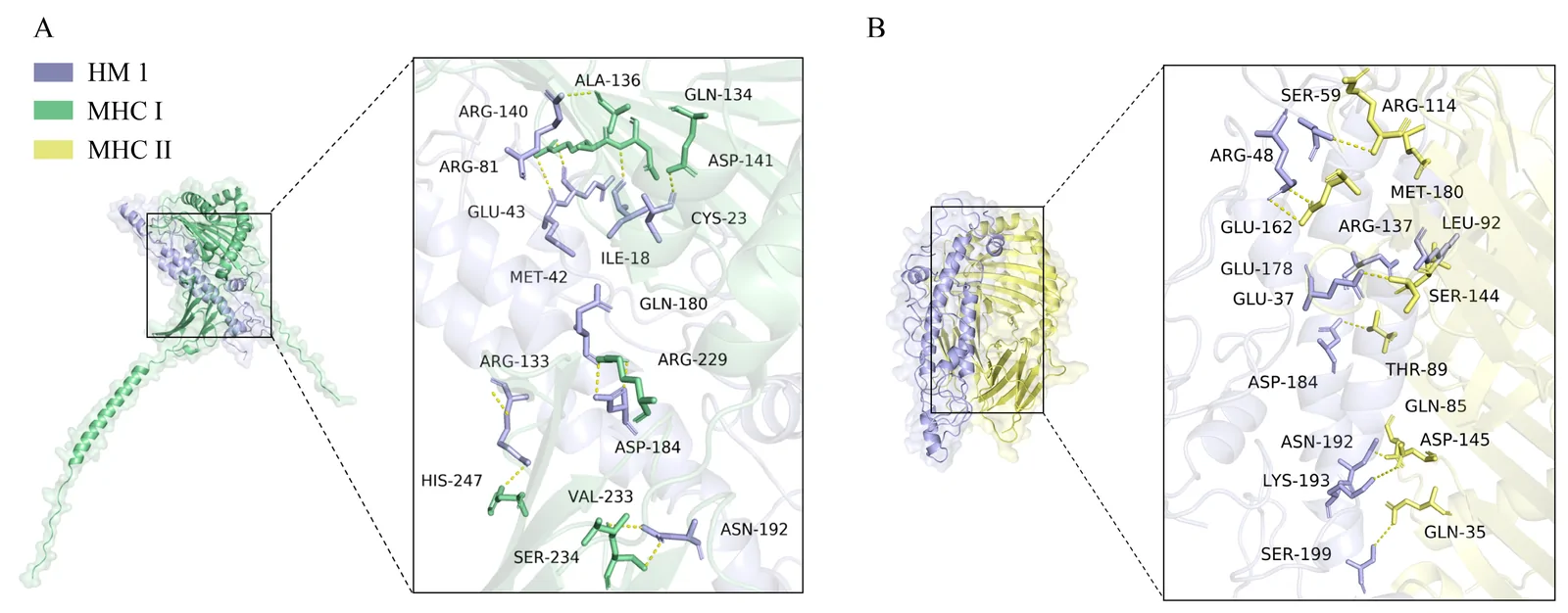
Molecular docking between HM1 and MHCs. (**A**) Diagram of the docking model of HM1-MHC I complex. (**B**) Diagram of the docking model of HM1-MHC II complex.

In order to assess the binding potential of the vaccine construct itself with innate immune receptors, molecular docking was performed using the GRAMM software to dock HM1 with TLR2 and TLR4, and the three-dimensional structures and interaction modes of the complexes were analyzed. Six docking models were generated for each complex, and the model with the highest energy score was selected as the optimal structure for subsequent analysis. Interface analysis of the optimal complexes was performed using PDBePISA. The results showed that the buried surface area of the HM1–TLR2 complex was 2441.4 Å^2^, the binding free energy was −7.1 kcal/mol, and it was stabilized by 10 hydrogen bonds and 13 salt bridges. In contrast, the HM1–TLR4 complex had a buried surface area of 2447.9 Å^2^, a binding free energy of −6.1 kcal/mol, and formed 14 hydrogen bonds and 15 salt bridges. Both complexes had hydrogen bonds and salt bridges widely distributed, suggesting a favorable binding affinity between HM1 and both receptors. To further characterize the non-covalent interactions at the binding interface, the HM1–TLR2 and HM1–TLR4 complexes were analyzed using COCOMAPS 2.0. The results showed that the interface of the HM1–TLR2 complex involved a total of 138 residues, which were stabilized by multiple non-covalent interactions, including 129 polar van der Waals contacts, 102 close contacts, 89 non-polar van der Waals contacts, 26 CH-O/N bonds, and one π-π, anion-π, and cation-π interaction each. The buried surface area was 2442.0 Å^2^, with the non-polar interface accounting for 55.27% and the polar interface accounting for 44.73%. The interface of the HM1–TLR4 complex involved a total of 132 residues, including 136 polar van der Waals contacts, 99 close contacts, 57 non-polar van der Waals contacts, 25 CH-O/N bonds, and one anion-π interaction. The buried surface area was 2447.25 Å^2^, with the non-polar interface accounting for 50.43% and the polar interface accounting for 49.57%. This indicates that the binding interfaces of HM1 with TLR2 and TLR4 are all stabilized by multiple non-covalent interactions. The three-dimensional structures of the docking complexes were visualized using PyMOL (Figure 6A and Figure 7A). To further identify the key amino acid residues involved in the binding interfaces, two-dimensional intermolecular interaction maps were generated using LigPlot+ (Figure 6B and Figure 7B). Additionally, to assess the binding potential of CTL and HTL epitopes in HM1 with antigen-presenting molecules, HM1 was docked with chicken MHC I and MHC II molecules. The results revealed that HM1 exhibited certain interactions with both MHC I and MHC II (Figure 8A,B), suggesting that it has the potential to be recognized by antigen-presenting molecules at the computational level. Overall, the molecular docking results indicated that HM1 exhibited certain binding affinities to TLR2, TLR4, and MHC molecules, providing preliminary structural evidence for its immunogenicity as a candidate vaccine.

### 3.7. iMODS-Based Normal Mode Analysis

**Figure 9:**
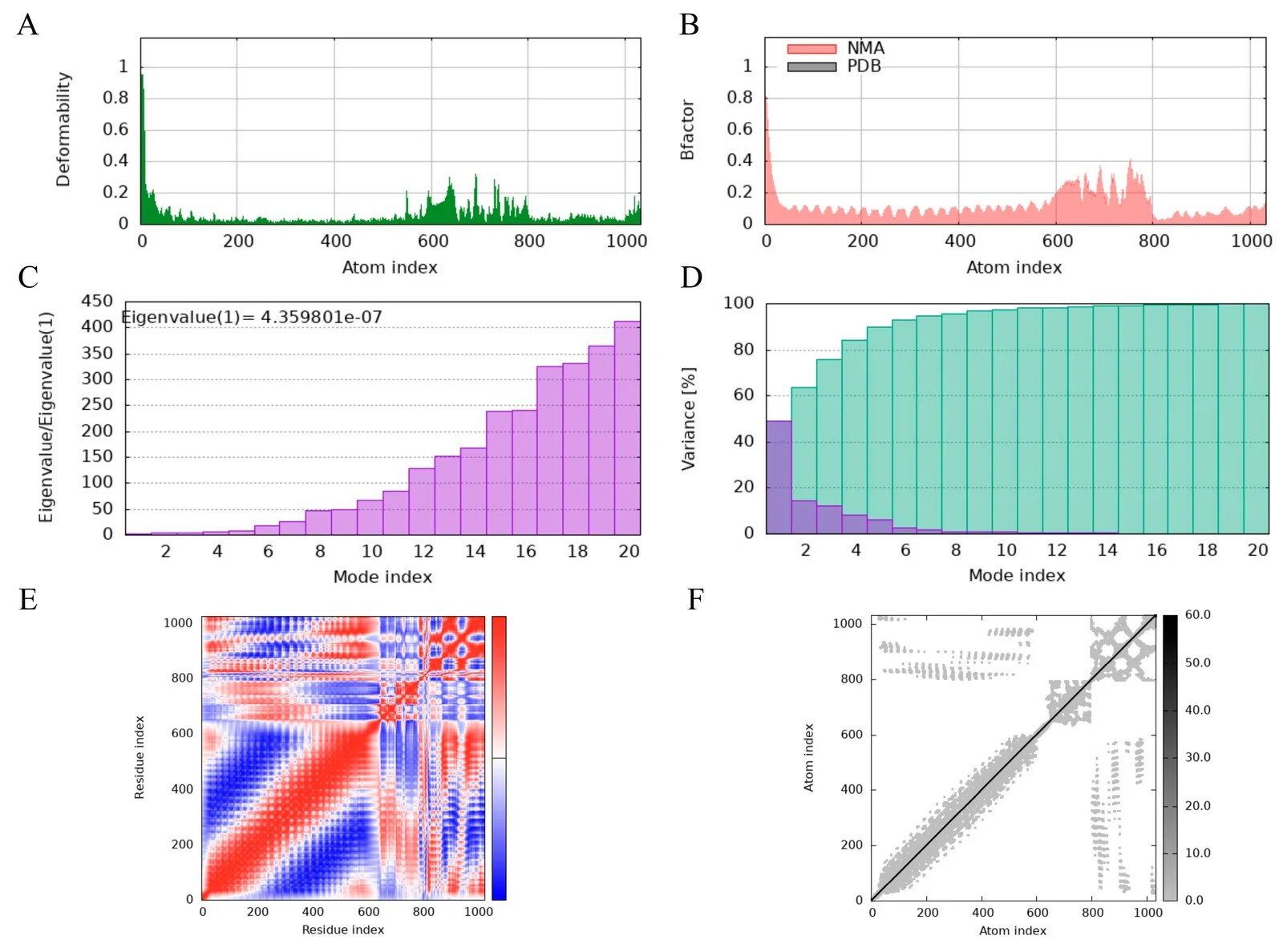
Normal mode analysis of HM1–TLR2 Complexes. (**A**) Deformability analysis plot. (**B**) B-factor analysis plot. (**C**) Eigenvalue distribution plot. (**D**) Structural variance plot. Individual (purple) and cumulative (green) variances were shown as colored bars. (**E**) Covariance plot. Matrix shows correlated (red), non-correlated (white), and anti-correlated (blue) motions of paired residues. (**F**) Elastic network model. Darker gray indicates stiffer regions.

To characterize the conformational dynamics properties of HM1–TLR2 complex, normal mode analysis was performed using iMODS. Atomic deformability analysis showed that the deformation ability of the N-terminal region of this complex is considerably higher than that of other regions, while the middle region showed overall low deformability with only local flexibility peaks observed in the 600–1000 atom interval (Figure 9A). The B-factor distribution was highly consistent with the deformability results, further supporting the regional flexibility differences in the complex (Figure 9B). The distribution of the first 20 low-frequency normal modes showed a stepwise increase in eigenvalues with increasing mode index. The first mode had an eigenvalue of 8.120376 × 10^−7^, which was the lowest among all modes and indicated that low-frequency modes dominated the large-scale collective motions of the complex (Figure 9C). Cumulative variance analysis demonstrated that the first 10 low-frequency modes could explain approximately 90% of the conformational variance, and the cumulative variance of the first 20 modes was close to 100%, indicating that the overall conformational changes in the complex were mainly dominated by a few low-frequency collective motions (Figure 9D). The analysis results of the residue movement correlation matrix indicate that there is a significant red coordinated movement area between the N-terminal and C-terminal residues, while the middle segment residues usually exhibit a low-correlation rigid characteristic, indicating that the dynamic behaviors of different regions of the complex have obvious domain-specificity (Figure 9E). The atomic distance fluctuation matrix showed that the majority of atomic distance fluctuations were concentrated near the matrix diagonal, with significant fluctuations only in the local regions outside the diagonal, suggesting that the overall conformation of the complex was based on a folded structure (Figure 9F).

**Figure 10:**
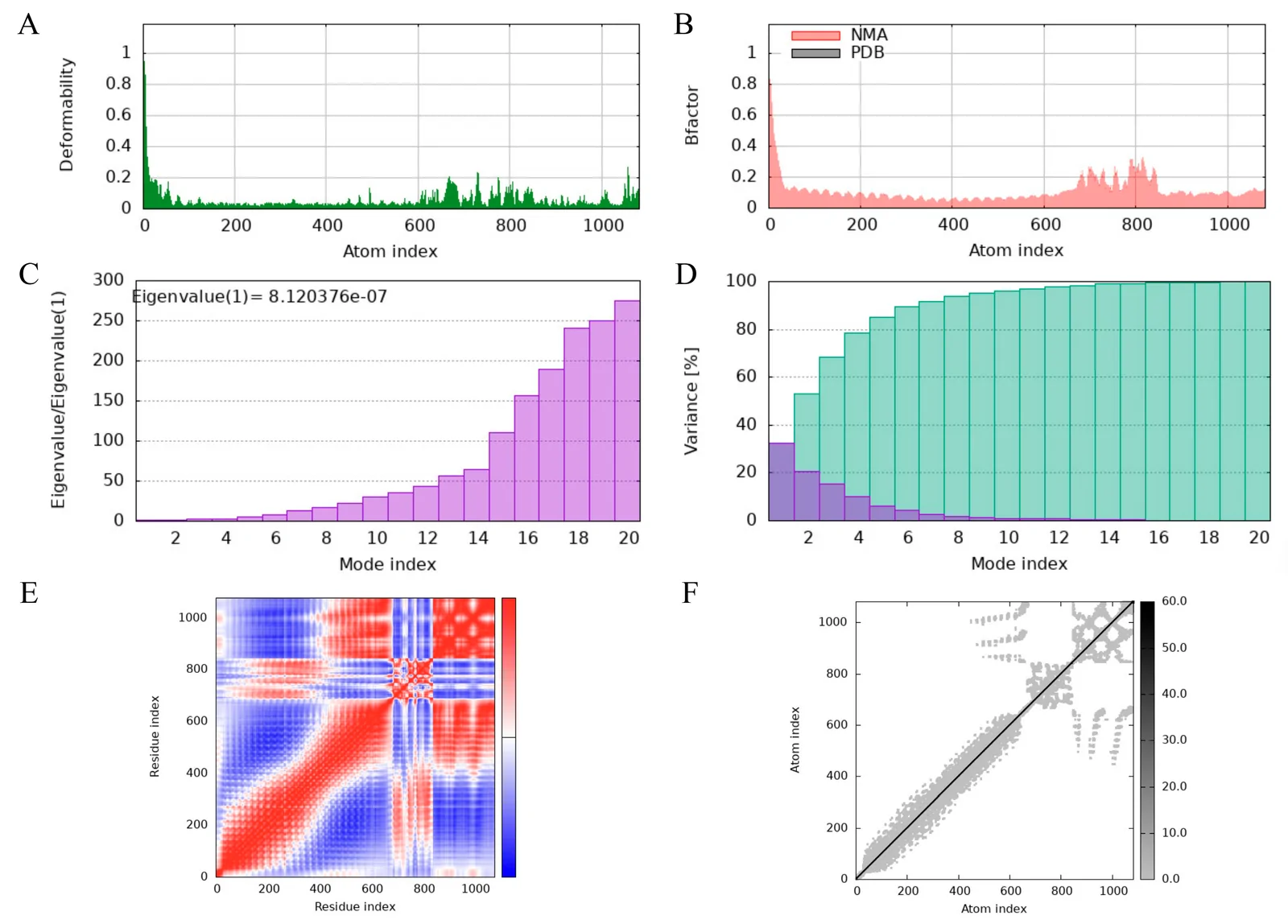
Normal mode analysis of HM1–TLR4 Complexes. (**A**) Deformability analysis plot. (**B**) B-factor analysis plot. (**C**) Eigenvalue distribution plot. (**D**) Structural variance plot. Individual (purple) and cumulative (green) variances were shown as colored bars. (**E**) Covariance plot. Matrix shows correlated (red), non-correlated (white), and anti-correlated (blue) motions of paired residues. (**F**) Elastic network model. Darker gray indicates stiffer regions.

The HM1–TLR4 complex exhibited a similar distribution pattern, characterized by high flexibility at the N-terminal and relatively rigid structure at the middle region. The normal mode characteristics and cumulative variance change trends were similar to those of the TLR2 complex, both dominated by low-frequency modes for overall conformational motion. The overall patterns of residue motion coupling and atomic distance fluctuation were similar, but there were certain differences in the positions of local flexibility peaks and the range of coordinated movement of residues (Figure 10).

### 3.8. Immune Simulation

**Figure 11:**
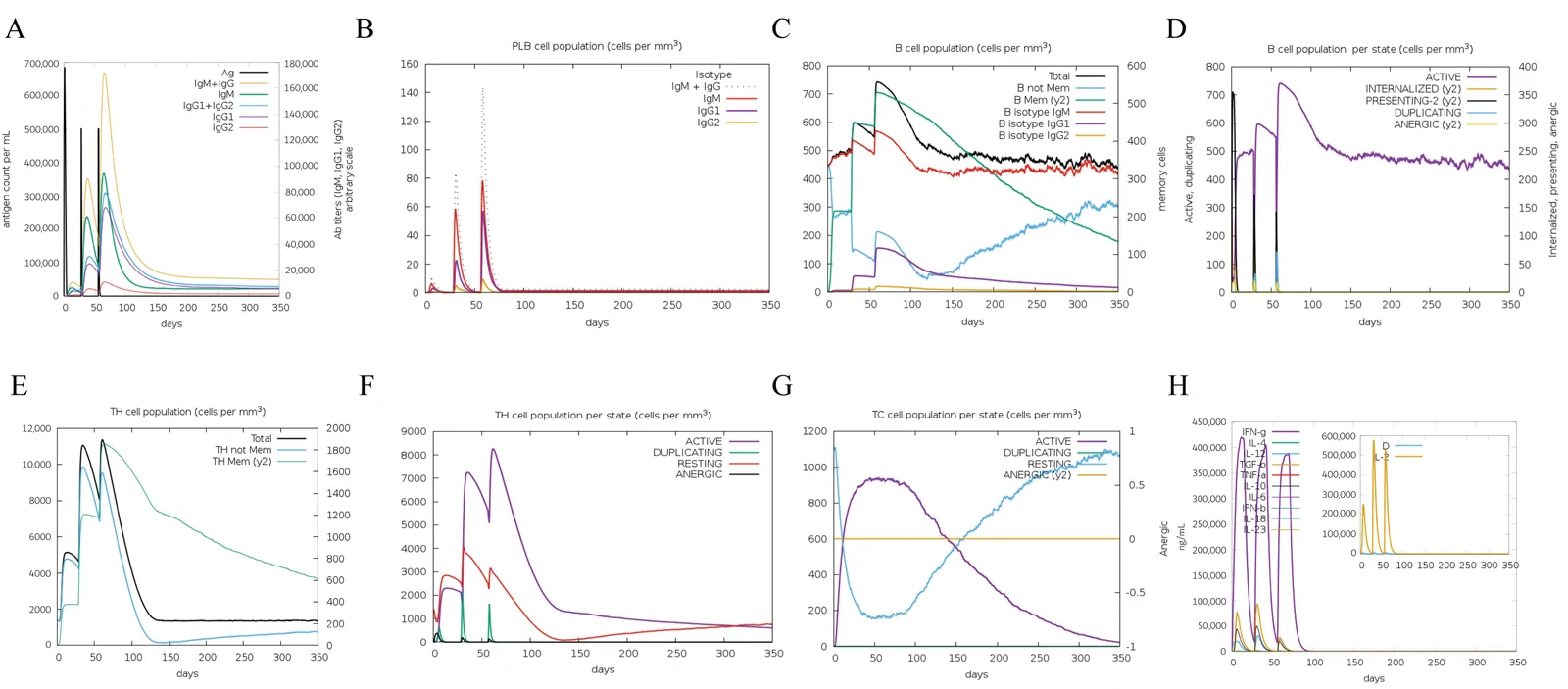
Immune simulation of HM1. (**A**) Immunoglobulin response against HM1. (**B**) Changes in PLB cell populations. (**C**) B cell population. (**D**) B cell population per state. (**E**) TH cell population. (**F**) TH cell population per state. (**G**) TC cell population per state. (**H**) Concentration of interleukins and cytokines.

The immune response elicited by HM1 was simulated and analyzed using the C-ImmSim. The results showed that after the second and third immunizations with HM1, the antibody titers of IgM + IgG, IgG1 + IgG2, and IgM showed an increasing trend (Figure 11A). The number of antibody-secreting cells of IgM, IgG1, and IgG2 reached a notable peak after the booster immunization (Figure 11B). The numbers of B subtype (IgM) and memory B cells exhibited marked increases (Figure 11C). Meanwhile, active B cells were predicted to be stimulated during the vaccination period (Figure 11D). During the simulation period, vaccination was also predicted to elicit total helper T (TH) cells and active TH cells (Figure 11E,F). The number of activated cytotoxic T (TC) cells was increased, while the number of resting TC cells showed a decreasing trend (Figure 11G). Furthermore, HM1 was predicted to induce higher levels of IFN-γ and IL-2 after each injection (Figure 11H). In summary, these simulation results suggest that HM1 has the potential to elicit strong humoral and cellular immune responses.

## 4. Discussion

Although H9N2 is classified as an LPAIV, its persistent prevalence and rapid evolution pose a serious threat to the global poultry industry. H9N2 infection mainly manifests as decreased production performance, immunosuppression and frequent secondary infections [32]. More critically, the RNA-dependent RNA polymerase of AIV lacks proofreading function, resulting in continuous accumulation of mutations during viral replication and making antigenic drift extremely likely to occur.

Traditional inactivated vaccines have significant limitations. Their immune protection is subtype-specific and mainly relies on humoral immunity, with limited ability to induce cellular immunity, resulting in severely insufficient cross-protection efficacy against heterologous strains. Unlike traditional vaccines that require complete pathogens or full-length proteins, MEV only screens and integrates the most critical immune advantage epitopes of the pathogen. This avoids the potential biological safety risks associated with complete pathogen cultivation. Moreover, by incorporating B-cell, CTL, and HTL epitopes simultaneously, MEV can simultaneously activate humoral immunity and cellular immunity [33]. Additionally, designing against conserved epitopes can also achieve broad-spectrum cross-protection against different variants.

As the primary surface antigen of AIV, the stem domain of HA2 in HA is highly conserved among different subtypes, and its sequence variation is significantly lower than that of HA1. HA2 plays a key role in mediating the fusion of the virus with the endosomal membrane [34]. Therefore, although the antibody titer against HA2 may be lower than that against HA1, its recognition spectrum is broader and can provide cross-protection against different subtypes and variants. M1, as one of the most conserved structural proteins of AIV, accounts for more than 30% of the total viral protein and plays an important role in the assembly and budding of the virus. M1 peptides can be efficiently presented by MHC class I molecules and are the main target antigens recognized by host CTLs. Previous studies have confirmed that M1-specific CD8^+^ T cell responses can recognize target cells infected with different subtypes of AIV and provide critical cross-protection when antibody levels are insufficient to neutralize the virus [35]. When HA2 and M1 are combined within the same vaccine construct, HA2 is responsible for inducing broad-spectrum neutralizing antibodies to block virus entry into host cells, while M1 is responsible for activating CTL responses to clear infected cells. This MEV design concept has been successfully applied in vaccine development against pathogens such as goatpox virus [36] and hepatitis C virus [37], and has been proven to effectively enhance cross-protection effects.

This study designed three different epitope combination H9N2 MEVs and conducted a systematic comparison. The proportion and layout of B-cell epitopes, CTL epitopes, and HTL epitopes may cause different biases in the immune response of the vaccine. HM1 contains 3 B-cell epitopes, 2 CTL epitopes, and 3 HTL epitopes, presenting a balanced layout of B/T cell co-recognition. HM2 incorporates more T-cell epitopes, thereby exhibiting a T-cell-biased profile. HM3 is primarily oriented toward humoral immunity. The secondary structure prediction results showed that the proportion of random coils and β-turns in HM1 is considerably higher than the other two schemes, indicating that the linear B-cell epitopes of HM1 have a higher exposure probability in the three-dimensional structure. B-cell epitopes can be recognized by antibodies only when they are located on the protein surface and have sufficient flexibility, and random coils can meet this requirement. The physicochemical property analysis further indicates that the instability index of HM1 (33.67) was lower than the stability threshold of 40, while HA2, M1, and HMf were all predicted to be unstable (>40), indicating that the rational connection of epitopes can improve the in vitro stability of the protein. The hydrophilicity index of HM1 (−0.52) was the lowest among all the compared proteins, indicating that it has better solubility. The isoelectric point (9.76) corresponds to a proportion of more than 65% of positively charged residues, indicating that HM1 is positively charged in a neutral physiological environment, which is conducive to electrostatic interaction with negatively charged cell membranes and may enhance the uptake efficiency of antigen-presenting cells for the vaccine.

The HM1 tertiary structure optimized by GalaxyRefine exhibits excellent stereochemical quality. The Ramachandran plot showed that 79.0% of the residues are located in the most favorable region, and the Z-score of ProSA-web is −4.98, which falls within the characteristic range of natural protein structures. This collectively indicated that the optimized HM1 model meets reliable model standards in both overall folding and local conformation, providing a reliable structural basis for subsequent molecular docking. TLR2 and TLR4 are key innate immune receptors for recognizing pathogen-associated molecular patterns, and their activation can induce the maturation of dendritic cells and the secretion of inflammatory cytokines. The molecular docking results showed that HM1 forms extensive intermolecular interaction networks with chicken TLR2 and TLR4. HM1–TLR2 complex is stabilized by 10 hydrogen bonds and 13 salt bridges, with a binding free energy of −7.1 kcal/mol. HM1–TLR4 complex forms 14 hydrogen bonds and 15 salt bridges, with a binding free energy of −6.1 kcal/mol, indicating that HM1 has the potential to be recognized and activated by TLR2 and TLR4 and to activate downstream signaling pathways. The COCOMAPS 2.0 analysis further revealed the molecular basis of the binding interface between HM1 and TLR2 and TLR4. The non-polar interfaces of both complexes accounted for more than 50%, indicating that hydrophobic interactions are the main driving force for the formation of the complexes. At the same time, a large number of polar van der Waals contacts and CH-O/N bonds provided additional specificity and cooperative stability for the interface. The docking results of HM1 with TLR receptors provide preliminary structural evidence for its immunogenicity. Additionally, the docking results between HM1 and chicken MHC I and MHC II molecules further suggest its CTL and HTL epitopes can be recognized by MHC molecules in silico. It should be noted that the molecular docking in this study employed GRAMM rigid-body docking, without considering the flexible conformational changes in the protein side chains.

Normal mode analysis revealed the dynamic behavior characteristics of the HM1–TLR complex. Atomic deformability analysis and B-factor distribution showed that the N-terminal region of the complex had higher conformational flexibility, while the middle region is relatively rigid. The high-flexibility regions facilitated induced-fit molecular recognition, while the rigid core maintains the overall rigidity of the complex. The first 10 low-frequency modes could explain approximately 90% of the variance in conformational fluctuations, indicating that the large-scale collective motion of the complex is mainly dominated by a few low-frequency modes, which is consistent with the dynamic characteristics of natural protein complexes. The residue movement-related matrix showed that there are cooperative movement regions between N-terminal and C-terminal residues, while the middle residues as a whole present low-related rigid characteristics, and the complex-bound interface exists correlated and anti-correlated motion patterns, which may play a key role in binding affinity and specific recognition. It should be noted that in this study, the TLR4 docking was performed using an extracellular monomer model and did not include MD-2. Given that TLR4 forms a complex with MD-2 under physiological conditions, this result cannot fully represent the receptor recognition process under physiological conditions. Moreover, this study did not conduct classical molecular dynamics simulations. The NMA results only reflect the intrinsic movement trend of the complex and cannot directly assess the binding stability. In conclusion, the above molecular docking and NMA analysis results are all predictions from computer simulations. Further verification is needed through the inclusion of the MD-2 model and molecular dynamics simulations in the future.

The C-ImmSim immune simulation results showed that HM1 can induce the secretion of IgM, IgG1 and IgG2 antibodies after three immunizations, and the numbers of active B cells, helper T cells and cytotoxic T cells all showed an upward trend. It is particularly noteworthy that the levels of IFN-γ and IL-2 are predicted to be upregulated. IFN-γ is the hallmark cytokine of Th1-type immune response and plays a core role in activating macrophages to clear viruses and promoting the differentiation of CTLs. IL-2 is a key driver for T cell proliferation and differentiation. The synergistic increase in these two cytokines indicated that HM1 may tend to induce Th1-biased immune responses, which is beneficial for eliminating virus-infected cells. Subsequent studies will validate the computer simulation predictions of this research through animal immune experiments and challenge protection tests.

## 5. Conclusions

This study successfully designed a broad-spectrum MEV candidate, HM1, targeting HA2 and M1 proteins of H9N2 using immunoinformatics methods. Systematic bioinformatics analysis revealed that HM1 has good physicochemical stability, high solubility, and strong antigenicity. The optimized tertiary structure meets the standards of a reliable model. Molecular docking and normal mode analysis predicted that HM1 can bind to chicken TLR2, TLR4, and MHC molecules in silico. Immune simulation indicated that it has the potential to induce both humoral and cellular immune responses simultaneously. This study provides new design ideas and a theoretical basis for addressing the technical challenges of H9N2 antigenic variation and weak cross-protection of existing vaccines, laying the foundation for subsequent vaccine construction and animal immune experiments.

## Figures and Tables

**Table 1:** Conserved sequences in screened HA2 and M1 proteins.

Protein Name	No.	Sequence Position	Amino Acids Length	Antigenicity
HA2	1	10–68	59	0.72
	2	70–138	69	0.64
	3	158–216	59	0.04
M1	1	1–36	36	0.14
	2	38–139	102	0.65
	3	159–221	62	0.83

**Table 2:** B-cell epitopes from HA2 protein of H9N2 were predicted using ABCpred.

No.	Sequence	Start Position	Conservation Rate (%)	Score
1-1	DSTQKAIDKITSKVNN	23	90	0.85
1-2	QHSNDQGVGMAADRDS	9	88	0.82
1-3	AGWYGFQHSNDQGVGM	3	94	0.79
1-4	GMAADRDSTQKAIDKI	17	90	0.77
1-5	DKMNKQYEIIDHEFSE	41	100	0.65
1-6	TSKVNNIVDKMNKQYE	33	96	0.61
2-1	LENQKTLDEHDANVNN	26	90	0.93
2-2	RALGSNAVEDGKGCFE	48	92	0.90
2-3	DDQIQDIWAYNAELLV	9	100	0.87
2-4	AVEDGKGCFELYHKCD	54	92	0.67

**Table 3:** CTL epitopes for M1 protein of H9N2 were predicted using NetMHCcons 1.1.

No.	Sequence	Start Position	Allele	Conservation Rate (%)	IC 50 (nM)
2-1	MEWIKTRPI	6	3	96	29.87
2-2	REMTFHGAK	68	3	100	361.90
2-3	KEVALSYST	76	3	100	130.88
3-1	HENRMVLAS	17	3	100	206.18

**Table 4:** HTL epitopes for M1 protein of H9N2 were predicted using NetMHCIIpan 4.3.

No.	Sequence	Start Position	Allele	Conservation Rate (%)	Rank (%)
2-1	KTRPILSPLTKGILGF	10	3	96	1.61
2-2	TRPILSPLTKGILGFV	11	3	96	1.61
2-3	RPILSPLTKGILGFVF	12	3	98	1.78
2-4	MDKAVKLYKKLKREM	56	2	100	0.37
2-5	DKAVKLYKKLKREMT	57	3	100	0.26
2-6	KAVKLYKKLKREMTF	58	2	100	1.33
3-1	TTNPLIRHENRMVLA	10	4	98	0.65
3-2	TNPLIRHENRMVLAS	11	4	100	0.43
3-3	NPLIRHENRMVLAST	12	4	100	0.40
3-4	PLIRHENRMVLASTT	13	3	100	2.20
3-5	AEAMEVASQARQMVQ	42	3	100	3.83
3-6	EAMEVASQARQMVQA	43	3	100	2.55
3-7	AMEVASQARQMVQAM	44	3	100	2.88

## Data Availability

The original contributions presented in this study are included in the article/Supplementary Material. Further inquiries can be directed to the corresponding authors.

## Supplementary Materials

The following supporting information can be downloaded at: https://www.mdpi.com/article/10.3390/microorganisms14081617/s1, Table S1. Background information of 50 selected virus strains used in this study.
