# Supplementary material for: Immunoinformatics Design of a Broad-Spectrum Multi-Epitope Vaccine Targeting HA2 and M1 of H9N2 AIV
Source: Microorganisms. 2026 Jul 24;14(8):1617. doi: 10.3390/microorganisms14081617 (PMC13515974; doi:10.3390/microorganisms14081617)
Supplement: Supplementary file 1 [file microorganisms-14-01617-s001.zip › microorganisms-4362836-supplementary.pdf]

Table S1 Background information of 50 selected virus strains used in this study.

Strains	HA GeneBank No.	M GeneBank No.	Collection year	Country of origin
A/turkey/California/189/66	AF156390.1	AF156471.1	1999	USA
A/turkey/Wisconsin/1/1966	CY130054.1	CY130055.1	1966	USA
A/chicken/Beijing/1/1994	KF188294.1	AF156466.1	1994	China
A/quail/Guangxi/B1/2006	EU086283.1	EU086285.1	2006	China
A/chicken/Yunnan/3511/2004	CY023896.1	CY023899.1	2004	China
A/chicken/Fujian/25/00	DQ064355.1	DQ064382.1	2000	China
A/Chicken/Shanghai/F/98	AF461532.1	AY253755.1	1998	China
A/chicken/Jiangsu/7/2002	FJ384759.1	FJ384746.1	2002	China
A/chicken/Hubei/C1/2007	EU365371.1	EU365374.1	2007	China
A/chicken/Henan/1.2/2008	FJ534538.1	FJ534541.1	2008	China
A/Chicken/Beijing/8/98	AF508562.1	AF508693.1	1998	China
A/Duck/Hong Kong/Y280/97	AF156376.1	AF156461.1	1997	China
A/chicken/Jilin/53/01	DQ064371.1	DQ064398.1	2001	China
A/chicken/Gansu/2/99	EF070733.1	EF070737.1	1999	China
A/chicken/Guangdong/47/01	DQ064359.1	DQ064386.1	2001	China
A/chicken/Shantou/3173/2001	CY024696.1	CY024699.1	2001	China
A/chicken/Shantou/5319/2001	CY024728.1	CY024731.1	2001	China
A/chicken/Guangdong/G7/2009	GU471897.1	KF367734.1	2009	China
A/chicken/HongKong/TP38/03	AY664670.1	KF188307.1	2003	China
A/chicken/Guangxi/55/2005	EU086245.1	EU086248.1	2005	China
A/environment/Hunan/1-70/2007	GU474566.1	GU474569.1	2007	China
A/chicken/Zhejiang/Q1D4/2010	JF715007.1	JN869526.1	2010	China
A/chicken/Yangzhou/11/2010	JN653617.1	JN653665.1	2010	China
A/Duck/Fujian/1753/2009	KP865944.1	KP866032.1	2009	China
A/chicken/Shandong/qd0307/2013	KM609578.1	KM609618.1	2013	China
A/chicken/Hubei/S1098/2015	MN647342.1	MN647345.1	2015	China
A/duck/Hunan/09.27_YYGKZ96-O/2016	MW108000.1	MW108003.1	2016	China
A/chicken/Hubei/S013/2017	MN647174.1	MN647177.1	2017	China
A/duck/Jiangxi/5.26_NCDZT83Q2-OC/2017	MW108856.1	MW108859.1	2017	China
A/chicken/Hubei/S1145/2016	MN647358.1	MN647361.1	2016	China
A/chicken/Guangdong/9.28_SZBJ018-O/2017	MW104192.1	MW104195.1	2017	China
A/chicken/Guangdong/7.18_SZBJ011-O/2018	MW104000.1	MW104003.1	2018	China
A/pigeon/Fujian/3.15_FZHX0009-C/2018	MW110304.1	MW110307.1	2018	China
A/chicken/Guangxi/8.25_NNXH007-O/2018	MW104240.1	MW104243.1	2018	China
A/chicken/Anhui Province/1220/2023	PQ899513.1	PQ899515.1	2023	China
A/chicken/Hubei/S340/2017	MN647390.1	MN647393.1	2017	China
A/chicken/Shanghai/15/2015	KU720441.1	KU720444.1	2015	China
A/chicken/Fujian/12.7_FZHX1213-O/2016	MW102904.1	MW102907.1	2016	China
A/duck/Fujian/3.16_FZHX0314-O/2017	MW107528.1	MW107531.1	2017	China
A/chicken/Guangdong/SIC38/2015	KX598461.1	KX598718.1	2015	China
A/chicken/Zhejiang/221/2016	KY056291.1	KY056312.1	2016	China
A/chicken/Guizhou/6.30_ZYLJJ005-O/2018	MW104424.1	MW104427.1	2018	China
A/chicken/Hubei/S462/2017	MN647414.1	MN647417.1	2017	China
A/duck/Hunan/06.22_YYGK79T3-OC/2018	MW107976.1	MW107979.1	2018	China
A/chicken/Hong Kong/G9/1997	KF188366.1	KF188369.1	1997	China
A/duck/Hong Kong/Y439/1997	KF188265.1	KF188268.1	1997	China
A/duck/Vietnam/OIE-2313/2009	AB545593.1	AB639355.1	2009	China
A/quail/Hong Kong/G1/1997	KY785896.1	KY785902.1	1997	China
A/Hong Kong/33982/2009	KF188316.1	KF188319.1	2009	China
A/turkey/Minnesota/38391-6/1995	LC431362.1	LC431365.1	1995	China
